# Supplementary material for: Patterns of Intron Gain and Loss in Fungi
Source: PLoS Biol. 2004 Nov 30;2(12):e422. doi: 10.1371/journal.pbio.0020422 (PMC532390; doi:10.1371/journal.pbio.0020422)
Supplement: Table S1 — Also available at http://genes.mit.edu/NielsenEtAl/. (4.3 MB ZIP). [file pbio.0020422.st001.zip › NielsenEtAl/html/784.html]

AN1003.1.NCU07697.1.MG01712.1.FG09580.1


```
 CLUSTAL W (1.82) Multiple Sequence Alignments - Introns Inserted


Sequence 1: AN1003.1	363 aa
Sequence 2: FG09580.1	381 aa
Sequence 3: NCU07697.1	379 aa
Sequence 4: MG01712.1	383 aa
Alignment Length: 383 aa
Number Identitical Residues: 272 aa
Alignment Score (without introns) 11238


MG01712.1 	MLALRRVAAAAPVARQFSAAPRAAATFAF~S0RYYSSQGERVAKFTGKKDAS0GRYPVSL
NCU07697.1	MLALRSIAAPAQR-QAFRAAPRAVSVSFQ0N~RFYSS---RVAQFTGQKDAS0GKYTVSL
FG09580.1 	MLAIRALRTPASRQALRTAAPRAA--IFY0N~RCYSTSGDRVAKYNGTKDAK0GNFLVSL
AN1003.1  	MFAARNFATPA-RQCLR------------~-~---ST---RVAPNLASTRLQ0GKYTVTL
          	*:* * . :.*                        *:   ***   . .  . *.: *:*

MG01712.1 	IEGDGIGPEISEAVKQIFEAAK0APVSWEPVDVTPILKDGRTAIPDAAIESIERNKVALK
NCU07697.1	IEGDGIGPEIAVAVKDIFAAAQ0TPINWEPINVDPILKDGKTAIPDAAIESIRRNKIALK
FG09580.1 	IEGDGIGPEIAQSVKDIFAAAK0TPIAWEPVDVTPIIKDGKTAIPDAAIDNIKKNKIALK
AN1003.1  	IEGDGIGPEISQSVKDIFSAAN0APIKWESVDVTPILKDGKTAIPDAAIDSVRKNYVALK
          	**********: :**:** **: :*: **.::* **:***:********:.:.:* :***

MG01712.1 	GPLA0TPIGKGHVSLNLTLRRTFNLFANLRPCRSVAGFKTPYDNVDTVLIRENTEGEYSG
NCU07697.1	GPLA0TPIGKGHVSLNLTLRRTFNLFANLRPCRSVAGFKTPYDGVDTVLIRENTEGEYSG
FG09580.1 	GPLA0TPIGKGHVSLNLTLRRTFNLFANLRPCRSVAGYETPYDNVNTVLIRENTEGEYSG
AN1003.1  	GPLA0TPVGKGHVSLNLTLRRTFNLFANLRPCRSVAGYKTPYDNVDTVLIRENTEGEYSG
          	**** **:*****************************::****.*:**************

MG01712.1 	IEHVVVDGVVQSIKLITREASERVLRFAFQHAQEIGRKKVRVVHKATIMKMSDGLFLSVG
NCU07697.1	IEHVVVDGVVQSIKLITREASERVLRYAFEQARAINRKKVRVVHKATIMKMSDGLFLNTA
FG09580.1 	IEHVVVDGVVQSIKLITREASERVLRFAFQHAESIGRKKVRVVHKATIMKLSDGLFLKVA
AN1003.1  	IEHVVVDGVVQSIKLITREASERVLRFAFQYARSINKKKVRVVHKATIMKMSDGLFLNTA
          	**************************:**: *. *.:*************:******...

MG01712.1 	QQVAKDFPGIEFDAELLDNTCLKMVTDPLPYNDKVLVMPNLYGDILSDMCAGLIGGLGLT
NCU07697.1	REVAQDFPDIEFDAELLDNTCLKMTTDPTPYNDKVLVMPNLYGDILSDMCAGLIGGLGLT
FG09580.1 	QEVAKDFPGIEFDAELLDNSCLKMVTDPTPYNDKVLVMPNLYGDILSDMCAGLIGGLGLT
AN1003.1  	REVAKDFPDVEFDAELLDNSCLKITTDPTPYNDKVLVMPNLYGDILSDMCAGLIGGLGLT
          	::**:***.:*********:***:.*** *******************************

MG01712.1 	PSGNIGDECSIFEAVHGSAPDIAGKGLANPTALLLSSMMMLRHMGLGEYADRIEKATFDT
NCU07697.1	PSGNIGDECSIFEAVHGSAPDIAGKGLANPTALLLSSMMMLRHMGLNEYADKIEKAAFDT
FG09580.1 	PSGNIGDECSIFEAVHGSAPDIAGKNLANPTALLLSSIMMLRHMGLNEHATRIEKAIFDT
AN1003.1  	PSGNIGDECSIFEAVHGSAPDIAGKGLANPTALLLSSIMMLQHMGLNEHASRIQKAIFDT
          	*************************.***********:***:****.*:* :*:** ***

MG01712.1 	LAEGK~ALTGDLGGKAKTHEFAGAIISKL
NCU07697.1	LAEGK~VLTGDLGGKASTKDFTSAIIDRL
FG09580.1 	LAEGK~ALTGDLGGKAKTNEYAAAIISRL
AN1003.1  	LAEGK0TLTGDLGGKAKTHEYADAIIKRL
          	***** .*********.*:::: ***.:*
```
